# Inhibition of Heat Shock Protein 90 Attenuates the Damage of Blood-Brain Barrier Integrity in Traumatic Brain Injury Mouse Model

**DOI:** 10.1155/2022/5585384

**Published:** 2022-04-12

**Authors:** Jia-ming Zhang, Yao Jing, Kun Wang, Jian-Tong Jiao, Jin-yu Xu, Jing Shi, Dong-dong Ji, Shou-rong Lu, Xiang-dong Li, Yun Zhang, Xiao-dong Cao

**Affiliations:** ^1^Department of Emergency, Wuxi People's Hospital Affiliated to Nanjing Medical University, Wuxi City, Jiangsu Province 214023, China; ^2^Department of Neurosurgery, Shanghai Jiao Tong University Affiliated Sixth People's Hospital, Shanghai Jiao Tong University, Shanghai 200233, China; ^3^Children's Surgical Center, The Affiliated Taian City Central Hospital of Qingdao University, Taian, Shandong 271000, China; ^4^Department of Neurosurgery, Wuxi People's Hospital Affiliated to Nanjing Medical University, Wuxi City, Jiangsu Province 214023, China; ^5^Department of Neurosurgery, First Affiliated Hospital of Soochow University, Soochow University, Soochow, Jiangsu Province 215000, China; ^6^Department of Nursing, Wuxi People's Hospital Affiliated to Nanjing Medical University, Wuxi City, Jiangsu Province 214023, China

## Abstract

Heat shock protein 90 (HSP90) is widely found in brain tissue. HSP90 inhibition has been proven to have neuroprotective effects on ischemic strokes. In order to study the role of HSP90 in traumatic brain injury (TBI), we carried out the present study. A novel inhibitor of the HSP90 protein, 17-dimethylaminoethylamino-17-demethoxygeldanamycin (17-DA), has been investigated for its function on the blood-brain barrier (BBB) damage after traumatic brain injury (TBI) in mouse models. These C57BL/6 mice were used as a TBI model and received 17-DA (0.1 mg/kg/d, intraperitoneally) until the experiment ended. To find out whether 17-DA may protect against TBI *in vitro*, bEnd.3 cells belonging to mouse brain microvascular endothelium were used. The HSP90 protein expressions were raised after TBI at the pericontusional area, especially at 3 d. Our study suggested that 17-DA-treated mice improved the recovery ability of neurological deficits and decreased brain edema, Evans blue extravasation, and the loss of tight junction proteins (TJPs) post-TBI. 17-DA significantly promoted cell proliferation and alleviated apoptosis by inhibiting the generation of intracellular reactive oxygen species (ROS) to downregulate cleaved caspase-3, matrix metallopeptidase- (MMP-) 2, MMP-9, and P-P65 in bEnd.3 cells after the injury. As a result, we assumed that the HSP90 protein was activated post-TBI, and inhibition of HSP90 protein reduced the disruption of BBB and improved the neurobehavioral scores in a mouse model of TBI through the action of 17-DA, which inhibited ROS generation and regulated MMP-2, MMP-9, NF-*κ*B, and caspase-associated pathways. Thus, blocking HSP90 protein may be a potential therapeutic strategy for TBI.

## 1. Introduction

Traumatic brain injury (TBI) is a worldwide health problem and a common reason for disability and death [1]. In the USA, there are approximately 5.3 million persons living with disabilities caused by TBI [2]. In China, the number of TBI is more than one hundred thousand in recent years [3]. These patients are either dead or suffer from permanent disability, and their families encounter huge economic challenges.

TBI is a continuous process of complex and dynamic pathophysiological changes and includes primary damage and secondary injury to the brain. Direct mechanical hurt to the brain leads to the primary damage tending to be unalterable. As a consequence of shock, a cascade of apoptosis, damage of the blood-brain barrier (BBB), cerebral edema, and oxidative stress are initiated, belonging to secondary injury [2]. There have been many studies aimed at minimizing secondary injury, but few can be worked on [4, 5].

The BBB act as an important functional barrier of the brain tissue. A major component of the BBB is the endothelial cells, which include the zonula occludens (ZO) and occludin as tight junction proteins (TJPs) [5]. After TBI, the BBB is broken because of the damage of continuous intercellular tight junctions (TJs) aggravating the secondary injury. Thus, the protection of BBB may be the novel therapeutic strategy to reduce secondary injury and further damage to the brain tissues.

Heat shock protein 90 (HSP90) belongs to the category of evolutionarily conserved and abundant heat shock protein which has been found to activate and stabilize more than 200 target proteins which are essential for normal physiological processes such as signal transduction, cell survival, and transcription [6]. In recent studies, the downregulation of HSP90 protein by the compound 17-dimethylaminoethylamino-17-demethoxygeldanamycin (17-DA) could slow down the development and damage of some diseases such as lung cancer [7], gastric cancer [8], and cerebral ischemic stroke [9]. In the present study, the effect of 17-DA, as the inhibitors of HSP90 protein, was investigated on mouse models for its potential to reduce the damage of BBB *in vivo* and *in vitro* post-TBI.

## 2. Materials and Methods

### 2.1. Animal Design

We prepared adult C57BL/6 mice (male, 8-10 weeks). Controlled cortical impact (CCI) of the mouse TBI model was created. After the mice were anesthetized with ketamine and thiazide, the heads of the mice were placed in a stereotactic frame. A midline incision of about 12 mm long above the head of mice was made under aseptic conditions. A trephine was used to expose a bone window with a diameter of 4 mm on the surface of the right parietal bone, 1 mm far from the sagittal suture. If the integrity of the dura mater was compromised, then the mice would be excluded from the study. The sham operation process had been completed. A CCI device (Needle-Precision Cortical Impactor PCI3000) was used for the craniocerebral injury model. A round steel impactor with a diameter of 3 mm was pressed lightly on the exposed intact dura, and it was used to hit the cortical surface at a vertical angle with 1.5 m/s impact velocity, 1.5 mm deformation depth, and 100 ms residence time. Sterile cotton was used to press the damaged cortical surface until the bleeding was controlled. 17-DA was purchased from Sigma and diluted with dimethyl sulfoxide (DMSO) and 0.9% sodium chloride (NaCl) solution. All mice were randomly divided into the sham operation group, the TBI group, and the 17-DA treatment group (0.1 mg/kg/d) for follow-up study.

### 2.2. Western Blot Analysis of Brain Tissue

The sample of brain tissues was taken from sham and TBI mice after 6 hours, 12 hours, 24 hours, 3 days, and 7 days and the sham, TBI+vehicle, and TBI+17-DA groups 3 days after TBI. The tissues were lysed in the mixed lysis buffer; the proteins from the brain tissue were separated using sodium dodecyl sulfate-polyacrylamide gel electrophoresis, and the same amount was loaded into the small well. Then, the brain tissue proteins were transferred into the polyvinylidene difluoride membrane, which was blocked by 5% sealing fluid, and incubated 12 hours to 16 hours at 4°C with the primary antibodies against HSP90, *β*-tubulin, GAPDH (1 : 1000; Abcam, UK), and TJPs (ZO-1 and occludin) (1 : 1000; CST, USA). After being washed, an appropriate amount of horseradish peroxidase-conjugated secondary antibodies (1 : 5000; CST, USA) were used to incubate with the membrane for 1 hour at room temperature. Protein markers were found on the gel imaging system (Millipore, USA) using a chemiluminescence reagent (Pierce, USA), and then, marker intensities were analyzed by the professional software (BioRad, USA).

### 2.3. Neurological Status Assessment

The modified neurological severity score (mNSS) was applied to determine the neurological deficit at 3 days after TBI in the injury control and treatment groups. The mNSS was calculated from 0 to 14. The higher the mNSS scores, the more severe was the neurological deficit which was found in the mouse models. The rotarod test was performed to judge the motor condition in mice. In a word, all the mice were trained with a speed from 4 to 40 rounds/min on the rod within 5 min for 3 days before TBI. After TBI, the time data of the rotarod test were recorded on the 3rd day.

### 2.4. Measurement of Brain Edema and Evans Blue (EB) Extravasation

The mouse head magnetic resonance imaging (MRI) was examined by the 3.0T scanner (EXCITE; Siemens Signa, USA) at TBI 3 days. The brain edema volume was assessed by the coronal T2-weighted scans. The extravasation of EB was used to assess the severity of BBB damage at TBI 3 days. The 2% EB dye was intravenously injected, and then, the mice were administered with 0.9% sodium chloride solution through heart perfusion to wash completely the intravascular dye 2 hours later. The mouse injured hemisphere was weighed immediately after the noninjured hemisphere was taken out. Then, they were homogenized in 50% trichloroacetic acid and centrifuged at 10,000 g for 15 min, then added 3 volumes of ethanol. EB contents of the samples were detected by the professional spectrophotometer (BioTek; USA) at a wavelength of 610 nm.

### 2.5. Cell Design

The bEnd.3 mouse brain capillary endothelial cells were cultured with a completed medium at 37°C in a humidified incubator. Mechanical stretch injury (SI) to bEnd.3 cells was done to create the TBI model *in vitro*. bEnd.3 cells were seeded into the special six-hole plate. After sufficient preparation, a biaxial SI on cells was created by the Cell Injury Controller II system (Virginia Commonwealth University, USA). All cells were divided into the control, SI, and 17-DA treatment groups (5 nM) for the following studies.

### 2.6. Cell Viability Assay

After SI, the cells were added vehicle and 17-DA, respectively, which were cultured for 24 hours. 10 *μ*l of the Cell Counting Kit-8 solution was mixed into the small well and then incubated for 2 hours. The absorbance of the sample at 450 nm was recorded by a professional spectrophotometer.

### 2.7. Assessment of Intracellular Reactive Oxygen Species (ROS)

Production of intracellular ROS was assessed using the fluorescent probe DCFH-DA (Beyotime Biotechnology, China). A 10 *μ*M DCFH-DA was added into different cell groups for 25 min in the dark at 37°C. After being washed, the samples were examined by the fluorescence spectrophotometer with 488 nm excitation wavelength and 535 nm emission wavelength.

### 2.8. Detection of Apoptosis

Apoptosis was assessed by flow cytometry using the specialized cell kit (Beyotime Biotechnology, China). In a word, the samples were added into 190 *μ*l of binding buffer and incubated in the dark at 25°C for 30 min. The apoptosis was examined by the professional flow cytometer.

### 2.9. Immunostaining

Cells were dealt with 4% paraformaldehyde and then penetrated into 0.1% TritonX-100. They were blocked by the professional sealing fluid. The cell samples were incubated with the primary antibody rabbit anti-cleaved caspase-3 (CST, USA) for 12 hours to 16 hours at 4°C. Subsequently, cells were added with anti-rabbit 488 secondary antibodies (1 : 500) in the dark for 1 hour. They were stained with DAPI (1 : 1000) in the dark for 10 min. The images were obtained from a professional microscope.

### 2.10. Western Blot Analysis of Cells

The cells were collected, and western blotting was performed on the brain tissues by use of the following primary antibodies: cleaved caspase-3, caspase-3, P-P65, and P65 (1 : 1000; CST, USA) and MMP-2, MMP-9, *β*-tubulin, and GAPDH (1 : 1000; Abcam, UK).

### 2.11. Statistical Analysis

All the data were performed by the mean ± SD. The one-way analysis was used to compare multiple groups, and then, differences between the two groups were examined by Student's *t*-test. *P* < 0.05 and *P* < 0.01 were considered statistically significant. Statistical analyses were conducted by the professional statistics software (SPSS Inc., USA). Quantified bar graphs were made by professional image software (GraphPad Software, USA).

## 3. Results

### 3.1. HSP90 Was Raised on the Pericontusional Area in TBI Mice

**Figure 1:**
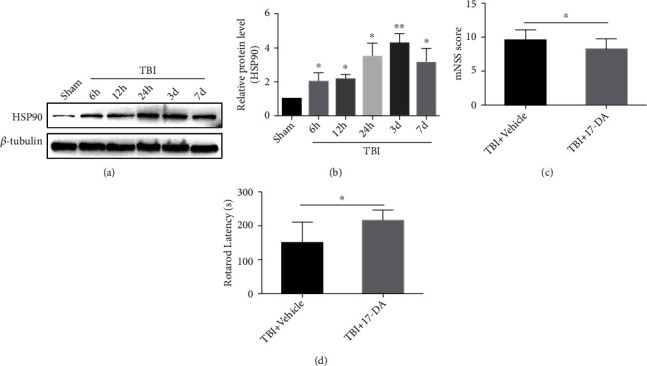
Expression of HSP90 in TBI mice before and after the injury and their neurological severity score. (a) HSP90 protein expression before and at different time points after TBI, as determined by western blotting. (b) Quantification of HSP90 levels from the immunoblotting experiment shown in (a). Data represent the mean ± SD (*n* = 3 per group). ^∗^*P* < 0.05 or ^∗∗^*P* < 0.01 vs. the sham group. The mNSS (c) and rotarod test (d) between the TBI+vehicle and TBI+17-DA groups (*n* = 12 per group). ^∗^*P* < 0.05.

The change levels of HSP90 protein post-TBI at different times were assessed by western blotting. The change in the level of HSP90 protein compared with the sham group was increased from 6 hours to 7 days after TBI and up to the peak at 3 days (*P* < 0.05 or *P* < 0.01) (Figures 1(a) and 1(b)).

### 3.2. 17-DA Improved the Recovery Ability of Neurological Deficits after TBI in Mice

It was observed that 3 days after TBI, the mNSS results of the 17-DA group were found to be lower than those of the vehicle group (*P* < 0.05) (Figure 1(c)). About the rotarod test, mice in the 17-DA group stayed on the rotarod longer on day 3 after TBI than in the vehicle group (*P* < 0.05) (Figure 1(d)).

### 3.3. 17-DA Alleviated Brain Edema through Protection of BBB after TBI in Mice

**Figure 2:**
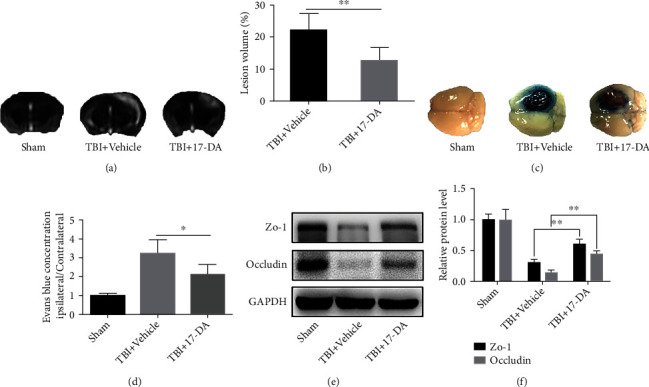
17-DA alleviated brain edema and damage of BBB and TJ proteins after TBI 3 days. (a, b) Representative T2-weighted images and quantified brain lesion levels were shown in different groups; the high signal on the injured side represented the edema area. (c, d) EB extravasations and quantified EB contents are shown. The blue color areas indicated extravasation of EB. (e, f) ZO-1 and occludin expressions in western blotting and quantified bar graph in different groups. Data were presented as the mean ± SD (*n* = 6 per group). ^∗^*P* < 0.05,  ^∗∗^*P* < 0.01.

The brain edema lesions were shown using high-intensity areas in the coronal T2-weighted MRI scans. Compared with the mice of the TBI+vehicle group, the lesion areas were smaller after being treated with 17-DA for 3 days post-TBI (*P* < 0.01) (Figures 2(a) and 2(b)).

The blue color represents the amount of EB extravasation at 3 days after TBI. The statistical analyses have shown that 17-DA treatment significantly reduced EB leakage (*P* < 0.05) (Figures 2(c) and 2(d)).

The expressions of ZO-1 and occludin were shown by western blotting in sham, TBI+vehicle, and TBI+17-DA. 17-DA treatment manifestly reduced the losses of TJ proteins (both *P* < 0.01) (Figures 2(e) and 2(f)).

### 3.4. 17-DA Promoted the Proliferation, Attenuated Intracellular ROS, and Decreased the Apoptosis in bEnd.3 Cells after SI

**Figure 3:**
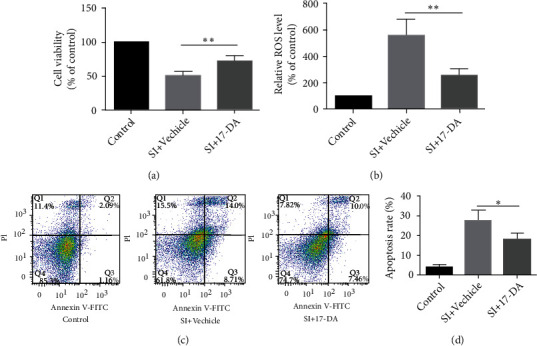
17-DA promoted the proliferation, attenuated intracellular ROS, and decreased the apoptosis after SI in bEnd.3 cells. (a) Bar graphs show cell viability after SI in different groups. (b) The intracellular ROS performed as the bar graph in three groups. (c, d) The situation of apoptosis by flow cytometry double-staining and bar graph analysis. Data were presented as the mean ± SD (*n* = 6 per group). ^∗^*P* < 0.05,  ^∗∗^*P* < 0.01.

CCK-8 assays were used to assess the bEnd.3 cell viability in three groups at 24 hours after SI. 17-DA obviously promotes cell proliferation in the SI+17-DA group compared with the SI+vehicle group (*P* < 0.01) (Figure 3(a)). At the same time, 17-DA inhibited the generation of intracellular ROS (*P* < 0.01) (Figure 3(b)) and decreased apoptosis (*P* < 0.05) (Figures 3(c) and 3(d)).

### 3.5. 17-DA Regulated Caspase-Associated, MMP-2, MMP-9, and NF-*κ*B Pathways in bEnd.3 Cells after SI

**Figure 4:**
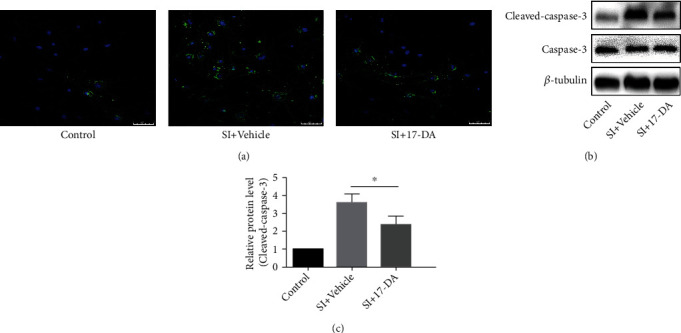
17-DA downregulated the expression of cleaved caspase-3. The levels of cleaved caspase-3 are shown by immunostaining (a) and western blotting (b). (c) Statistical bar graph for (b). Data were presented as the mean ± SD (*n* = 6 per group). ^∗^*P* < 0.05.

**Figure 5:**
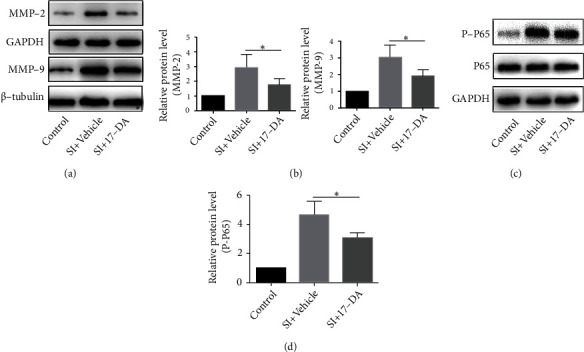
17-DA downregulated the expressions of MMP-2, MMP-9, and P-P65. (a, c) Western blotting shows the levels of MMP-2, MMP-9, and P-P65/P65. (b, d) Statistical bar graph performed. Data were presented as the mean ± SD (n = 6 per group). ∗P < 0.05.

Cleaved caspase-3 was regarded as having a close relationship with apoptosis. We found that 17-DA significantly reduced the activation of caspase-3 by immunostaining and western blotting (*P* < 0.05) (Figures 4(a)–4(c)). Meanwhile, the levels of MMP-2, MMP-9, and P-P65/P65 were downregulated by the treatment of 17-DA after SI in bEnd.3 cells (both *P* < 0.05) (Figures 5(a)–5(d)).

## 4. Discussion

TBI is regarded as a public health problem in modern-day society leading to disability and death [10]. Many TBI animal models that simulate human TBI conditions were investigated to examine the potential of drug therapy. They included lateral fluid percussion brain injury [11], weight-drop brain injury [12], blast brain injury [13], and penetrating brain injury [14]. The CCI model was adopted in this study because of its high accuracy, which allowed us to estimate the numerical impact value to create different levels of pathological TBI in the study. In our experiments, the animal models having moderate brain injury were used.

We demonstrated that the changes in HSP90 caused by TBI were consistent with the literature reports in cerebral ischemic stroke [9]. As an analog of geldanamycin, 17-DA is an inhibitor of HSP90 protein. Previous studies have examined 17-DA's antiapoptosis, anti-inflammatory, antioxidative stress, and anticancer effects [7–9, 15]. This compound (17-DA) was used in our study for the protection of BBB after TBI.

As an active window between the central nervous system and peripheral blood circulation, the BBB had two functions: firstly, it prevented the entry of harmful substances into the brain from the blood, and secondly, it also regulates the transport of nutrients and metabolic wastes in and out of the brain [16, 17]. The damage of BBB was often found after TBI, cerebral ischemic stroke, and so on [18] which led to the formation of cerebral edema. A greater degree of BBB damage was associated with more cerebral edema. 17-DA reduced the cerebral edema through attenuating the disruption of TJs in the BBB after TBI. At the same time, the mNSS and rotarod test were used to assess the neurological functional recovery including motor, sensory, balance, and reflex after TBI in mice [19]. Obviously, inhibition of HSP90 improved the neurobehavioral function in TBI mice.

To explore the protection mechanism of HSP90 on the brain cells, cell experiments were performed. The experiment was performed using the mouse brain endothelial bEnd.3 cells as an injury model in *in vitro* studies [20]. In our study, we detected that 17-DA promoted proliferation and decreased apoptosis in bEnd.3 cells after SI. ROS including superoxide, the hydroxyl radical, hydrogen peroxide, and hypochlorous acid was the major production of oxidative stress caused by TBI [21]. ROS may attack DNA, proteins, membrane lipids, and transcription factors and activate several signaling pathways such as caspase-3, NF-*κ*B, MMP-2, and MMP-9 [22, 23]. The increase in ROS after a TBI was closely associated with mitochondrial damage [17]. The released cytochrome c after mitochondrial damage promoted the caspase-3 activation. The cleaved caspase-3 plays a crucial role and carries out apoptosis [24, 25]. After the injury to the brain, NF-*κ*B was known to play a critical role in the pathology of neuroinflammation. Activation of p65 NF-*κ*B in the cytoplasm and phosphorylation by oxidative stress led to its entry into the nucleus, where it promoted the release of inflammatory factors and induced apoptosis [26]. MMP-2 and MMP-9 were considered to be important proteins that interfered with the leakage of the BBB. They were produced in the cell in primary forms and were activated by cleaving the propeptide after being released into the extracellular space. The release of MMP-2 and MMP-9 accelerated the formation of cerebral edema [27, 28].

We had two limitations to our study. It was believed that cerebral edema was caused by damage to the BBB. Many scientists, however, argue that cytotoxic edema is an important component of brain edema [29]. However, in this study, cytotoxic edema was not explored. As for the HSP90, it should have an effect on several signaling pathways. Hence, in the future, more research will be conducted to explore the effect of HSP90 on various signaling pathways.

## 5. Conclusions

In summary, our findings demonstrated that 17-DA inhibited disruption of BBB which reduced brain edema in TBI mice and attenuated loss of TJs, contributing to improved recovery ability of neurological deficits. It has been proposed that 17-DA reduces intracellular ROS generation to regulate MMP-2, MMP-9, NF-*κ*B, and caspase-associated pathways. Hsp90 inhibition may be a therapeutic strategy for TBI.

## Data Availability

The data used to support the findings of this study are available from the corresponding authors upon request.
